# Cascade Sliding Mode Control for Linear Displacement Positioning of a Quadrotor

**DOI:** 10.3390/s25030883

**Published:** 2025-01-31

**Authors:** Albert Sawiński, Piotr Chudzik, Karol Tatar

**Affiliations:** Institute of Automatic Control, Faculty of Electrical, Electronic, Computer and Control Engineering, Lodz University of Technology, 90-924 Łódź, Poland; albert.sawinski@dokt.p.lodz.pl (A.S.); piotr.chudzik@p.lodz.pl (P.C.)

**Keywords:** sliding mode control, quadcopter, positioning, cascading system, simulation tests, modeling

## Abstract

This paper contains an example of a simulation implementation of sliding mode control algorithms for the problem of adjusting the linear position of a quadrotor. A mathematical model of the drone was proposed, which was then implemented in a simulation environment. The method of designing sliding mode controllers using the Lyapunov method in order to improve stability was presented. A cascade system based entirely on sliding mode control algorithms is introduced. The article ends with a comparative analysis of simulation test results of classical control systems and controllers based on sliding mode control.

## 1. Introduction

The quadrotor aircraft belongs to the category of Unmanned Aerial Vehicles (UAVs), commonly referred to as drones [1]. The growing popularity of these devices in professional applications has contributed to intensive technological development. The requirements for the quality of drone performance are constantly being expanded, especially those related to increasing their resistance to interference caused by changes in wind direction, strong gusts caused by explosions and contact with obstacles. This can be even more visible in linear positioning tasks, where the stability and precision of the position adjustment are particularly important. Currently, more and more often, as in many other disciplines that require complex control algorithms or where regulation processes are difficult to implement, attempts are being made to use artificial intelligence methods. Artificial intelligence algorithms work very well for controlling both a single UAV and entire formations of drones, where interference is caused not only by changeable external conditions (e.g., wind) but also by communication disturbances [2,3,4]. However, it is possible to achieve good results in the classical way. One of the promising directions in the development of drone control techniques is the use of sliding mode control (SMC) methods, which give much better results than classic methods based on PID controllers [5,6,7,8,9,10,11,12,13]. This work required the development of a mathematical model of a quadrotor, which is a rather complicated and strongly nonlinear object, and conducting simulation studies enabling the synthesis of appropriate control systems. As part of the work, two types of control systems (linear and nonlinear) for drone positioning were designed to allow for testing their operation in the aforementioned simulation environment. First, the system was adapted to control angular positions, which is necessary for the further construction of a cascade system with master linear positioning controllers. For both outer and inner control loops, the performances of two mentioned methods (PID and SMC) were compared. Studies of the operation of selected control methods allowed for obtaining information about the quality of control, the impact of disturbances on the operation of the system and the impact of partial unawareness of the system parameters or their inaccuracy. The most important aspect of this work is the comparative analysis of classical control methods with sliding mode control algorithms, especially due to the presence of the abovementioned difficulties and those implemented within the modeled system. The target sliding mode control system proposed in this study pertains to a cascaded structure of sliding mode controllers that closely cooperate based on the principle defined by the binding two-stage equivalent control law. One of the primary requirements for this controller was the ability to mitigate the phenomenon known as chattering, which is a typical issue encountered in traditional sliding mode control approaches.

## 2. Quadrotor Model

A quadrotor is an object with complex dynamics, characterized by strong nonlinearity, the impact of which is particularly evident as the drone’s speed increases [5,6,7,8,9,14,15,16,17]. The design of the control systems of a quadrotor should take into account that it is an object with six degrees of freedom (DOF), which are represented by three axes of linear motion and three Euler angles. The object has four control quantities, which are successive lifting forces of the individual rotors [5,6,7,8,10,17,18,19].

**Figure 1:**
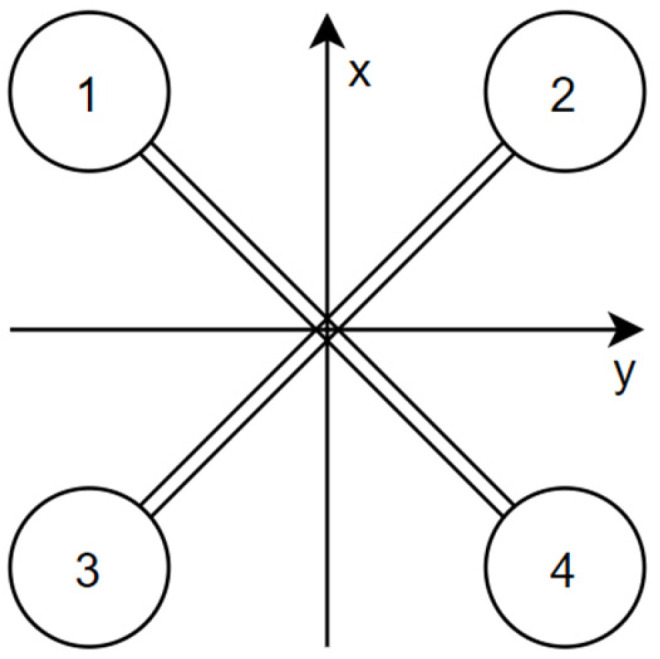
The “X” type structure of a quadrotor with the axes described.

Figure 1 represents the structure of a quadrotor type “X” with the adopted rotor numbering. The graphic also shows the orientation of the coordinate system and the directions of the X and Y axes. Each of the four rotors is capable of generating lift directed along the Z axis [20]:

(1) $$\left[\begin{array}{c} F_{x}\\ F_{y}\\ F_{z} \end{array}\right]=\left[\begin{array}{c} 0\\ 0\\ F_{1}+F_{2}+F_{3}+F_{4} \end{array}\right],$$

where

- $F_{1}$, $F_{2}$, $F_{3}$, $F_{4}$—lift forces generated by successive drone motors;
- $F_{x}$, $F_{y}$, $F_{z}$—forces acting in the three axes of the global reference system.

Thus, it is possible to generate lift only directed along the *Z*-axis of the local drone system [7,10,14,16,18,21]. The drone is not able to generate forces in the X and Y axes of the local system, so linear motion in these axes is carried out by obtaining the appropriate orientation. Taking this into account, after the transformation from the local to the global system frame reference, the following set of equations is obtained:

(2) $$\left[\begin{array}{c} F_{zx}\\ F_{zy}\\ F_{zz} \end{array}\right]=\left[\begin{array}{c} F_{z}\left(c\alpha s\theta c\varphi +s\alpha s\varphi \right)\\ F_{z}(c\alpha s\theta s\varphi -s\alpha s\varphi )\\ F_{z}(c\alpha c\theta ) \end{array}\right],$$

where

- $F_{zx}$, $F_{zy}$, $F_{zz}$—projections of lift acting in the Z axis of the local system onto individual axes of the global system;
- c, s—shortened notation: cosine and sine;
- α, θ, φ—three Euler angles: roll, pitch and yaw.

The dependence of lift force and three torques on the individual rotational speeds of the rotors is described as follows:

(3) $$\left\{\begin{array}{c} F_{z}=\;\sum_{i\;=1}^{4}F_{i}=b({\omega_{1}}^{2}+{\omega_{2}}^{2}+{\omega_{3}}^{2}+{\omega_{4}}^{2})\\ \tau_{\alpha }=bl({\omega_{1}}^{2}-{\omega_{2}}^{2}+{\omega_{3}}^{2}-{\omega_{4}}^{2})\\ \tau_{\theta }=bl({\omega_{1}}^{2}+{\omega_{2}}^{2}-{\omega_{3}}^{2}-{\omega_{4}}^{2})\\ \tau_{\varphi }=d\left({\omega_{1}}^{2}-{\omega_{2}}^{2}-{\omega_{3}}^{2}+{\omega_{4}}^{2}\right), \end{array}\right.$$

where

- $\omega_{1}$, $\omega_{2}$, $\omega_{3}$, $\omega_{4}$—rotational speeds of the respective motors;
- d—torque resistance coefficient with respect to the Z axis;
- l—arm length;
- $\tau_{\alpha }$, $\tau_{\theta }$, $\tau_{\varphi }$—individual torques;
- b—the drag coefficient of the drone, which determines the ratio of the rotational speed of the motor to the force it generates determined by the formula $b=\frac{F_{i}}{\omega_{i}^{2}}$.

The dependence of individual torques on the corresponding angular accelerations can be described by the following system of equations [7,8,11]:

(4) $$\left\{\begin{array}{c} \ddot{\alpha }=\frac{\tau_{\alpha }+\dot{\theta }\dot{\varphi }(I_{yy}-I_{zz})}{I_{xx}}\\ \ddot{\theta }=\frac{\tau_{\theta }+\dot{\alpha }\dot{\varphi }(I_{zz}-I_{xx})}{I_{yy}}\\ \ddot{\varphi }=\frac{\tau_{\varphi }+\dot{\alpha }\dot{\theta }(I_{xx}-I_{yy})}{I_{zz}}, \end{array}\right.$$

where

- $\ddot{\alpha }$, $\ddot{\theta }$, $\ddot{\varphi }$—individual angular accelerations;
- $\dot{\alpha }$, $\dot{\theta }$, $\dot{\varphi }$—individual angular velocities;
- $I_{xx}$, $I_{yy}$, $I_{zz}$—moments of inertia around individual axes.

In a similar form, it is possible to write the dependence of individual linear accelerations on the resultant lift force [7,9,11]:

(5) $$\left\{\begin{array}{c} \ddot{x}=\frac{F_{z}}{m}\cdot \left(s\alpha s\varphi +c\alpha s\theta c\varphi \right)-T_{x}\\ \ddot{y}=\frac{F_{z}}{m}\cdot \left(c\alpha s\theta s\varphi -s\alpha c\theta \right)-T_{y}\\ \ddot{z}=\frac{F_{z}}{m}\cdot c\alpha c\theta -g-T_{z}, \end{array}\right.$$

where

- $\ddot{x}$, $\ddot{y}$, $\ddot{z}$—individual linear accelerations;
- m—mass of the drone;
- g—acceleration due to gravity;
- $T_{x}$, $T_{y}$, $T_{z}$—dynamic resistance of motion in individual axes.

Aerodynamical drag, on the other hand, depends on the respective angular velocities and are determined by the following equations:

(6) $$\left\{\begin{array}{c} T_{x}=\;k_{Tx}\dot{x,}\\ T_{y}=k_{Ty}\dot{y},\\ T_{z}=k_{Tz}\dot{z}, \end{array}\right.$$

where

- $\dot{x}$, $\dot{y}$, $\dot{z}$—linear velocities in individual axes;
- $k_{Tx}$, $k_{Ty}$, $k_{Tz}$—coefficients of dynamic drag in individual axes.

The maximum torques can be determined using the following formulas:

(7) $$\tau_{m_{roll,pitch}}=2bl\omega_{m}^{2},$$

(8) $$\tau_{m_{yaw}}=2d\omega_{m}^{2},$$

where

- $\tau_{m_{roll,pitch}}$—maximum torque relative to the Y and X axis;
- $\tau_{m_{yaw}}$—maximum torque relative to the Z axis.

The most important quantities that affect the dynamics of the drone are the mass of the drone, the individual moments of inertia around the respective axes of the reference frame relative to the drone frame, and the lift forces generated by the four motors.

## 3. Implementation of the Classic Quadrotor Angular Positions Control System

A quadrotor drone is an object with six degrees of freedom. It is possible to determine its orientation using three Euler angles—roll, pitch and yaw—as well as its position in space on the X, Y and Z axes. Thus, the system has more degrees of freedom than controlled quantities. During the mathematical modeling of the quadrotor drone, two reference frames were used. The first is a coordinate system oriented to the ground, while the second is a coordinate system oriented to the drone itself. Conversion from one system to another is possible by using appropriate rotation matrices. Taking into account both of these systems allows for obtaining information about linear and angular positions relative to the ground and about the required displacements relative to the drone itself in order to obtain the set values. The purpose of the control is to achieve the preset values of the angular positions of the drone and its altitude.

**Figure 2:**
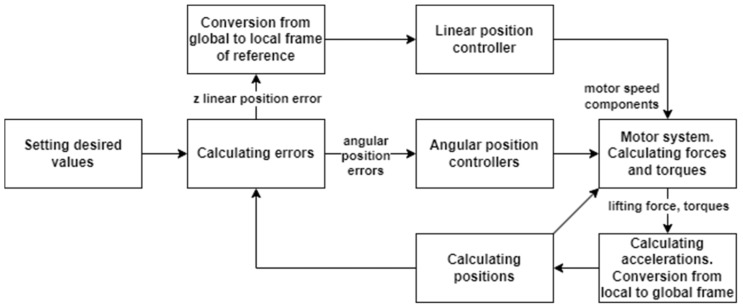
Proposed control system schematic diagram for angular positioning.

In the conducted research, the system control system of angular positions was used (Figure 2) [22]. This system consists of four controllers, and each of them is responsible for control of one of four positions: three angles and altitude. This system is the basis for the development of a linear positioning system. The conversion from the global to the local system applies only to the error of the linear position in the Z axis.

Used as a classical solution, PID controllers are one of the most popular and widely known control methods due to their uncomplicated structure and ease of implementation [22,23]. The model uses built-in elements that implement continuous PID controllers. A control system consisting of PID controllers without proper tuning can prove unreliable in the case of a highly nonlinear control object, especially in the presence of interference [7].

The parameters of the proportional, integrating, and differentiating parts were selected using an empirical method, i.e., manual tuning [7,19].

## 4. Synthesis of Sliding Mode Controllers for a Quadrotor Angular Positioning System

The sliding control method is Variable Structure Control (VSC) [24] or Variable Structure System (VSS) [25,26]. This boils down to the use of different feedbacks that act on opposite sides of a certain switch hyperplane in the state space [24]. Therefore, it is possible to design such a control system that the operating point of the object (determined by the values of the state variable vectors) remains on it after reaching the coordinates to which a certain hyperplane belongs, and moves along it in a sliding motion [24]. In the case where this point moves only on a certain hyperplane, its motion is called a perfect sliding motion [24]. The form of the hyperplane depends on the order of the object; for example, for an object of the second order, it will be a line of switches, while for an object of the third order it will be a plane, etc. [24,27]. The process of regulation by this method consists of two stages [24,27]:

- The reaching phase, during which the point representing the state of the object moves from the initial coordinates to a certain hyperplane, and the object is susceptible to disturbances and the influence of model imperfections.
- The sliding phase, in which the mentioned point moves in a hyperplane, trying to reduce error values to zero, and the object becomes robust. Reaching this phase means that the dynamics of the object are described only by the parameters of this hyperplane, and the order of the object is lowered.

The main advantage of sliding mode control is its high robustness to all kinds of interference and to the influence of inaccuracies of the model of the controlled object [24,27]. This has also been confirmed in the case of the quadrotor object control system [5,8,9,10,12,13,15,17]. SMC controllers can be successfully used in the case of nonlinear objects where there are uncertainties in knowing the full dynamics [24]. In the case of an object that is a quadrotor characterized by strong nonlinearity, the use of nonlinear controllers should increase the stability of the system and reduce the impact of interference and imperfections of the model [5]. Due to the application of the same principles of sliding mode control design, a vector notation was used for each of the angular positions in order to describe all control laws at the same time. A vector of sliding variables $s_{\eta }$ using the errors for each of the angular positions [8,13,15,28] has been proposed, which has the following form:

(9) $$s_{\eta }=\left({\dot{\eta }}_{d}-\dot{\eta }\right)+\Lambda_{\eta }\left(\eta_{d}-\eta \right),$$

where

- $\eta_{d}$—vector of desired angular positions—$\left[\alpha_{d},\;\theta_{d},\;\varphi_{d}\right]$;
- η—vector of angular positions—$\left[\alpha ,\;\theta ,\;\varphi \right]$;
- $\Lambda_{\eta }$—direction coefficients vector of the sliding variables for altitude control—[$\lambda_{\alpha },\;\lambda_{\theta },\;\lambda_{\varphi }$];
- $s_{\eta }$—vector of angular sliding variables—[$s_{\alpha }$, $s_{\theta }$, $s_{\varphi }$].

The equivalent part of the control law can be determined by comparing the derivative of the sliding variable to zero [8,10,27,29] (for discontinuous control equal to zero):

(10) $$\dot{s_{\eta }}=\left({\ddot{\eta }}_{d}-\ddot{\eta }\right)+\Lambda \left({\dot{\eta }}_{d}-\dot{\eta }\right).$$

If an appropriate matrix is proposed, which contains the corresponding elements of the system of equations marked with the number 4:

(11) $$M=\left[\begin{array}{c} \dot{\theta }\dot{\varphi }(I_{yy}-I_{zz})\\ \dot{\alpha }\dot{\varphi }(I_{zz}-I_{xx})\\ \dot{\alpha }\dot{\theta }(I_{xx}-I_{yy}) \end{array}\right],$$

where

- M—matrix proposed to simplify the determination of the equivalent control vector.

This Equation (10) can be transformed into the following form [8]:

(12) $$\dot{s_{\eta }}={-I}^{-1}\left[M+u_{eq}\right]+{\ddot{\eta }}_{d}+\Lambda \dot{e}.$$

Thus, after comparing the derivative of the vector of sliding variables to zero, the equivalent control form is obtained:

(13) $$u_{eq}=I\left({\ddot{\eta }}_{d}+\Lambda \dot{e}\right)+M.$$

Subsequently, the form of discontinuous control was adopted [8]:

(14) $$u_{D}=asgn\left(s_{\eta }\right),$$

where

- a—vector of gain of control signals—$\left[a_{\alpha },\;a_{\theta },\;a_{\varphi }\right]$.

In order for the control system to be able to reduce the error to zero, it is necessary to meet the condition that the product of the sliding variable and its derivative is less than zero. Testing the stability of a sliding mode control for the drone system can be performed using the appropriate Lyapunov function [8,10,15,17,20,26,30]. It is also important that the gain of the discontinuous control is greater than the maximum possible interference gain [8,24,26]. In order to investigate the stability of the system, the following Lyapunov function has been proposed [15,20]:

(15) $$V=\;\frac{1}{2}{s_{\eta }}^{T}s_{\eta },$$

where

- V—Lyapunov function.

A form of the system derivative of this Lyapunov function was obtained:

(16) $$\dot{V}=\;{s_{\eta }}^{T}\dot{s_{\eta }}={s_{\eta }}^{T}({-I}^{-1}\left[M+u\right]+{\ddot{\eta }}_{d}+\Lambda \dot{e}).$$

The control signal is treated as equal to the signal $u_{eq}$, which results in the following relation:

(17) $$\dot{V}=\;{s_{\eta }}^{T}\dot{s_{\eta }}=0.$$

If, on the other hand, the control signal is the sum of the equivalent and discontinuous control, then the derivative will take the following form:

(18) $$\dot{V}=\;{s_{\eta }}^{T}\dot{s_{\eta }}=-\frac{{s_{\eta }}^{T}a\mu sgn\left(s_{\eta }\right)}{I}.$$

Thus, it follows from Equation (18) that for positive gain vectors of discontinuous control, the value of the system derivative of the Lyapunov function is less than zero:

(19) $$\dot{V}=\;{s_{\eta }}^{T}\dot{s_{\eta }}<0.$$

The system is therefore asymptotically stable, and the error should tend to zero. Analogously, to the laws of angular position control, a sliding variable for the linear position in the Z axis has been proposed.

(20) $$s_{z}={\dot{e}}_{z}+\lambda_{z}e_{z},$$

where

- $s_{z}$—sliding variable for control of the altitude of the drone;
- $\lambda_{z}$—direction coefficient of the sliding variable in case of altitude control;
- $e_{z}$—error of linear position control in the Z axis.

Then, the derivative of this sliding variable was determined:

(21) $${\dot{s}}_{z}=\left({\ddot{z}}_{d}-\ddot{z}\right)+\;\lambda_{z}{\dot{e}}_{z}.$$

The proposed control law takes the following form:

(22) $$u_{z}=\;u_{zeq}+u_{zD},$$

where

- $u_{z}$—control signal of the linear position controller in the Z axis;
- $u_{zeq}$—signal of the equivalent part of the control;
- $u_{zD}$—signal of the discontinuous part of the control.

By comparing the derivative of the sliding variable and the value of the discontinuous control to zero, the equivalent control was obtained:

(23) $$u_{zeq}=\;\frac{m}{\cos\left(\alpha \right)\cos\left(\theta \right)}\left(T_{z}+g+{\ddot{z}}_{d}+\lambda_{z}{\dot{e}}_{z}\right).$$

The form of discontinuous control was adopted, as in the case of positioning angular positions:

(24) $$u_{zD}=a_{z}sgn\left(s_{z}\right),$$

where

- $a_{z}$—positive sliding variable coefficient.

In order to investigate the stability of the system, the proposed form of the Lyapunov function was used again:

(25) $$V_{z}=\frac{1}{2}s_{z}^{2},$$

where

- $V_{z}$—Lyapunov function.

The system derivative of this Lyapunov function is determined:

(26) $${\dot{V}}_{z}=s_{z}{\dot{s}}_{z}=s_{z}\left(\left({\ddot{z}}_{d}-\ddot{z}\right)+\;\lambda_{z}{\dot{e}}_{z}\right).$$

For the case where $u_{z}=u_{zeq}$, the following relation is obtained:

(27) $${\dot{V}}_{z}=s_{z}{\dot{s}}_{z}=0.$$

On the other hand, for the case from Equation (24), the following were obtained:

(28) $${\dot{V}}_{z}=s_{z}{\dot{s}}_{z}=-\frac{\cos\left(\alpha \right)\cos\left(\theta \right)s_{z}a_{z}sgn(s_{z})}{m}.$$

So, if the $a_{z}>0$ and angular positions of the roll and pitch are in the range (−π; π), then,

(29) $${\dot{V}}_{z}=s_{z}{\dot{s}}_{z}<0.$$

Equation (29) shows that the system is asymptotically stable. If one of the roll or pitch angles reaches a value of −π or π, this derivative would be zero, so the system would not be asymptotically stable. On the basis of Equations (9)–(29) presented in this subsection, a model of the angular position control system based on SMC controllers was implemented in the simulation environment.

## 5. Simulation Tests of the Implemented Angular Positioning Systems

Tests performed in the simulation environment included a presence of the disturbances. Tests were carried out for both PID controllers and sliding mode control algorithms. Natural external disturbance acting on the object of a quadrotor under conditions of outdoor environment is wind, the modeling of which often refers to the operation of wind turbines [30]. In this paper, a simplified model of disturbance is proposed, which is expressed in newton-meters and directly affects torques [8]. Analogously to the classic control method, also for the SMC-based system, simulation data were collected, and appropriate waveforms were generated. An additional interference is the delay of the measurement loop of the drone’s angular position signals, which is 0.001 s.

The first stage of each of the tests carried out was to set a positive value of the linear Z position of the drone. Then, the values of the set angular positions were changed using appropriate signals. The target result was a situation in which the drone changes its one angular position and then returns to its initial position. This process should be repeated in turn for each angle. The final stage of the tests involved changing all three angular positions at the same time and then returning to the initial position.

**Figure 3:**
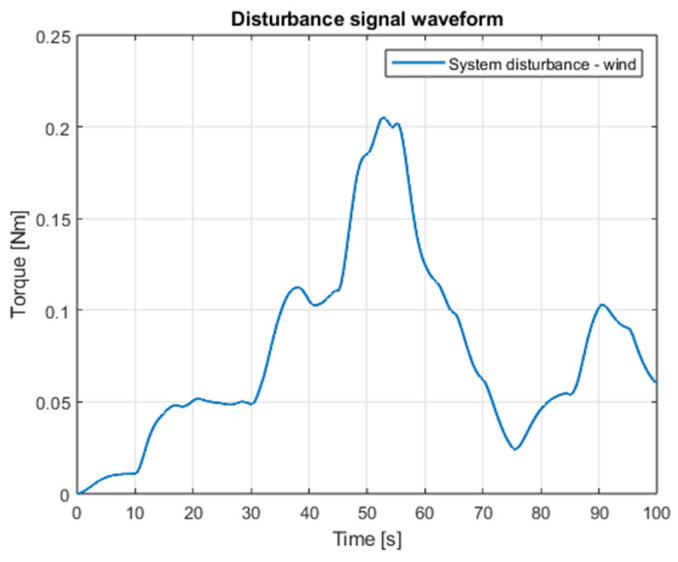
The waveform of the modeled external interference signal.

**Figure 4:**
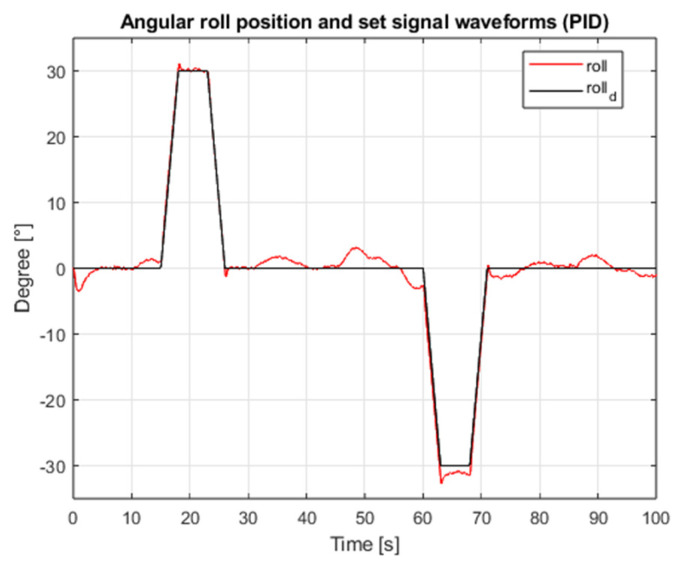
Waveforms of the angular position of the roll and the setpoint value (PID, interference gain: 1, measurement delay: 0.001 s).

**Figure 5:**
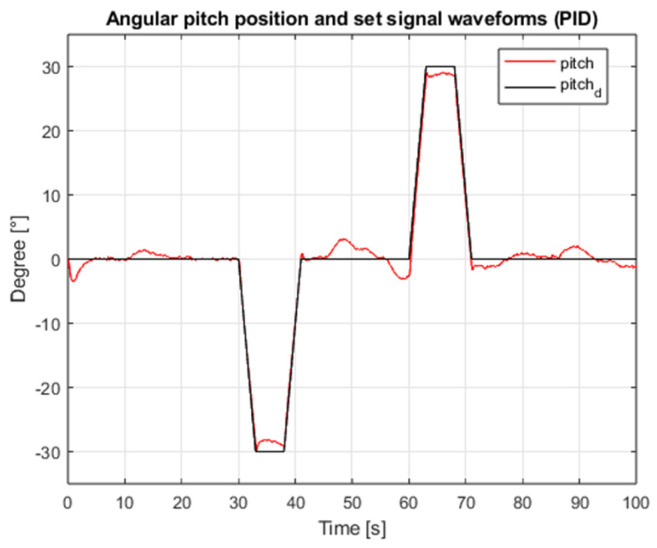
Waveforms of pitch angular position and setpoint (PID, interference gain: 1, measurement delay: 0.001 s).

**Figure 6:**
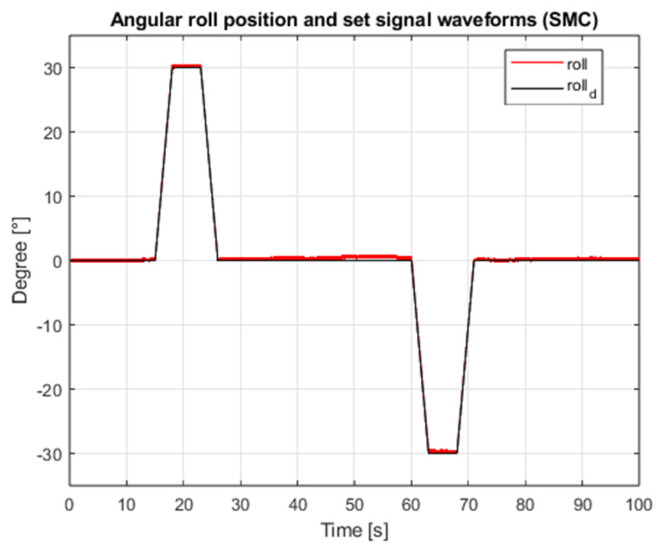
Waveforms of the angular position of the roll and the setpoint value (SMC, interference gain: 1, measurement delay: 0.001 s).

**Figure 7:**
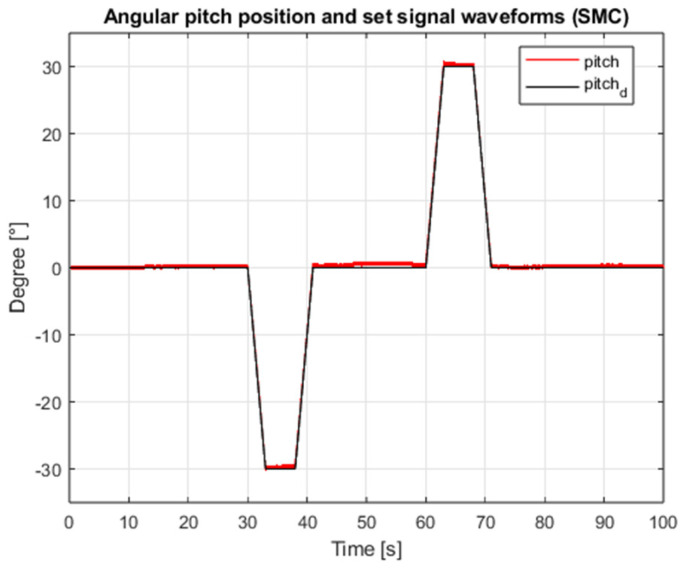
Waveforms of pitch angular position and setpoint (SMC, interference gain: 1, measurement delay: 0.001 s).

Figure 3 shows the waveform of the proposed external interference signal. The results of the simulation for the case of both angular position controllers are shown in Figure 4, Figure 5, Figure 6 and Figure 7, which present angular position signals and set signals. The presented results indicate that the use of sliding mode control algorithms in the case of controlling an object affected by strong external disturbances allows for reduction in the impact of these disturbances to a significant extent; overshoots caused by the applied external torque were limited. Detailed data and their analysis are presented in Section 9.

## 6. Implementation of the Classic Linear Positioning Outer Loop Control System

Since there are fewer controlled variables than the output variables of the drone, and it is not possible to directly generate forces directed in accordance with the X and Y axes of the local reference frame relative to the drone’s propulsion system, the linear positioning of the drone in these axes is obtained indirectly [9,11,17,20,26]. Hence, the form of the linear position control system is slightly more complicated than in the case of the first, abovementioned angular positioning system, namely, it is necessary to use two superior linear position controllers in the X and Y axes, which affect the input signal of the slave angular position controllers, pitch and roll, respectively. The second structure of the control system therefore consists of six controllers. In each of the two cases, the controllers related to the linear position in the Z axis and the angular position yaw are not elements of the cascade control. The first cascade system implemented is a system containing PID controllers as the outer loop.

**Figure 8:**
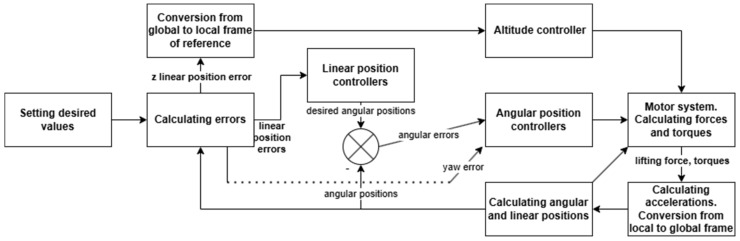
Proposed control system schematic diagram for linear positioning.

Figure 8 shows a simplified diagram of the linear control system of the quadrotor. The linear controller in the Z axis and the yaw angular controller remain unchanged, and an additional element is the master controller’s system for X and Y linear positioning. The pitch and roll controllers’ main purpose is to calculate proper control signals in the inner loop.

Two different linear positioning systems are discussed: those based on classical controllers and sliding mode control algorithms. The latter, in this case, means as follows: in the first system, all controllers are based on PID control, and in the second system, all controllers are based on sliding mode control algorithms.

## 7. Synthesis of Sliding Mode Controllers for a Linear Positioning Outer Loop Control System

In order to design linear positioning controllers in the outer loop of the control system, which are based on sliding mode control algorithms, another analysis was carried out. Again, it started with proposing an appropriate sliding variable. The presented case concerns the *X*-axis positioning algorithm, and for the *Y*-axis, the SMC controller design procedure should be carried out in an analogous way. The proposed form of the new sliding variable:

(30) $$s_{x}={\dot{e}}_{x}+\lambda_{x}e_{x},$$

where

- $s_{x}$—sliding variable for control of the *X*-axis position of the drone;
- $\lambda_{x}$—direction coefficient of the switching line for *X*-axis position control;
- $e_{x}$—error of linear position control in the Z axis.

Then, the derivative of this sliding variable was determined:

(31) $${\dot{s}}_{x}=\left({\ddot{x}}_{d}-\ddot{x}\right)+\;\lambda_{x}\dot{e_{x}}.$$

The proposed control law takes the following form:

(32) $$u_{x}=\;u_{xeq}+u_{xD},$$

where

- $u_{x}$—control signal of the linear position controller in the Z axis;
- $u_{xeq}$—signal of the equivalent part of the control;
- $u_{xD}$—signal of the discontinuous part of the control.

Again, to determine the equivalent control, the derivative of the sliding variable and the discontinuous control are compared to zero. Additionally, signal determination is based on the system of Equation (5):

(33) $${\dot{s}}_{x}=0\;\Rightarrow \;\ddot{x}={\ddot{x}}_{d}+\;\lambda_{x}{\dot{e}}_{x},$$

substituting from the system of Equation (5):

(34) $$\;\frac{F_{z}}{m}\cdot \left(s\alpha s\varphi +c\alpha s\theta c\varphi \right)-T_{x}={\ddot{x}}_{d}+\;\lambda_{x}{\dot{e}}_{x}.$$

In the discussed case, the linear position controller’s main purpose is to generate desired value of the proper angular position signal. Based on this information, it is possible to assume that, in this case,

(35) $$\alpha =\alpha_{d}=u_{xeq},$$

which leads to the following form:

(36) $$s\alpha_{d}s\varphi +c\alpha_{d}s\theta c\varphi =\frac{\left({\ddot{x}}_{d}+\;\lambda_{x}{\dot{e}}_{x}+T_{x}\right)m}{F_{z}}.$$

As the force in the Z axis, we assume the equivalent control signal generated by the altitude controller (Equation (23)):

(37) $$s\alpha_{d}s\varphi +c\alpha_{d}s\theta c\varphi =\frac{\left({\ddot{x}}_{d}+\;\lambda_{x}{\dot{e}}_{x}+T_{x}\right)m}{\frac{m}{c\alpha_{d}c\theta }\left(T_{z}+g+{\ddot{z}}_{d}+\lambda_{z}{\dot{e}}_{z}\right)}.$$

Based on this, the following equation was obtained:

(38) $$\frac{s\alpha_{d}s\varphi +c\alpha_{d}s\theta c\varphi }{c\alpha_{d}c\theta }=\frac{\left({\ddot{x}}_{d}+\;\lambda_{x}{\dot{e}}_{x}+T_{x}\right)}{\left(T_{z}+g+{\ddot{z}}_{d}+\lambda_{z}{\dot{e}}_{z}\right)},$$

which allows for obtaining the following:

(39) $$tan\left(\alpha_{d}\right)=\frac{\left(\frac{{\ddot{x}}_{d}+\;\lambda_{x}{\dot{e}}_{x}+T_{x}}{T_{z}+g+{\ddot{z}}_{d}+\lambda_{z}{\dot{e}}_{z}}-tan\left(\theta \right)c\varphi \right)c\theta }{s\varphi }.$$

Finally, it is possible to obtain the following equivalent control law, for φ≠kπ,k∈Z:

(40) $$\alpha_{d}=u_{xeq}=arctan\left(\frac{\left(\frac{{\ddot{x}}_{d}+\;\lambda_{x}{\dot{e}}_{x}+T_{x}}{T_{z}+g+{\ddot{z}}_{d}+\lambda_{z}{\dot{e}}_{z}}-tan\left(\theta \right)c\varphi \right)c\theta }{s\varphi }\right).$$

The form of modified discontinuous control contains a variable amplitude, which allows for chattering phenomena impact reduction and improved control quality [13,25,31,32,33,34,35]:

(41) $$u_{xD}={{\left|s_{x}\right|}^{\mu }a}_{x}sgn\left(s_{x}\right),$$

where

- $a_{x}$—positive sliding variable coefficient;
- μ—power exponent parameter for discontinuous variable gain.

The following form of the Lyapunov function is proposed to investigate the stability of the discussed system:

(42) $$V_{x}=\frac{1}{2}s_{x}^{2},$$

where

- $V_{x}$—Lyapunov function.

The system derivative of this Lyapunov function is determined:

(43) $${\dot{V}}_{x}=s_{x}{\dot{s}}_{x}=s_{x}\left(\left({\ddot{x}}_{d}-\ddot{x}\right)+\;\lambda_{x}{\dot{e}}_{x}\right).$$

For the case where $u_{x}=u_{xeq}$, the following relation is obtained:

(44) $${\dot{V}}_{x}=s_{x}{\dot{s}}_{x}=0.$$

Hence, the system is stable in the sense of Lyapunov. As mentioned earlier, a similar analysis was conducted for the *Y*-axis position case.

## 8. Simulation Tests of the Implemented Linear Positioning Systems

Tests of the linear positioning control systems were performed in a similar manner to the angular positioning case. The same disturbance signal was applied to the system (Figure 3). A proposed control task could be described as an attempt to perform a flight of the quadcopter in a previously precisely planned movement trajectory.

**Figure 9:**
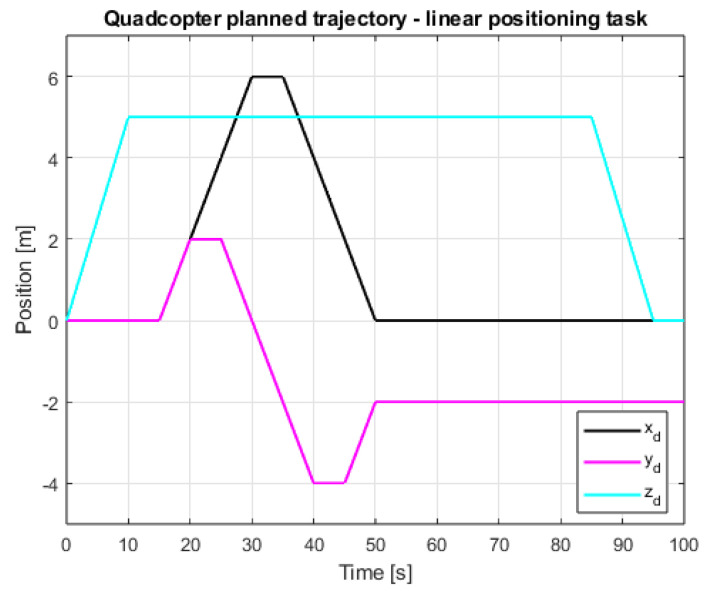
Planned linear trajectory of the quadcopter movement—2D view.

**Figure 10:**
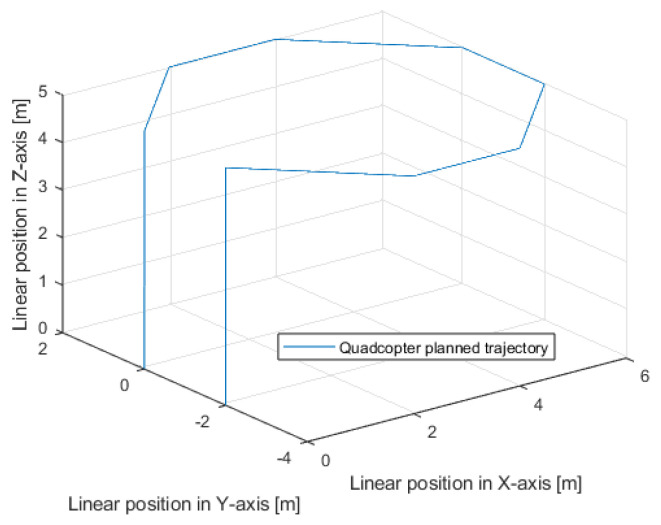
Planned linear trajectory of the quadcopter movement—3D view.

In Figure 9 and Figure 10, the same quadcopter desired trajectory is visible. In order to enable the quadcopter to maneuver freely in the X and Y axes, it is necessary for it to reach a certain set altitude at very beginning of the test; hence, the drone takes off at the beginning of the test and lands at the very end, after completing the planned movements in the other axes.

**Figure 11:**
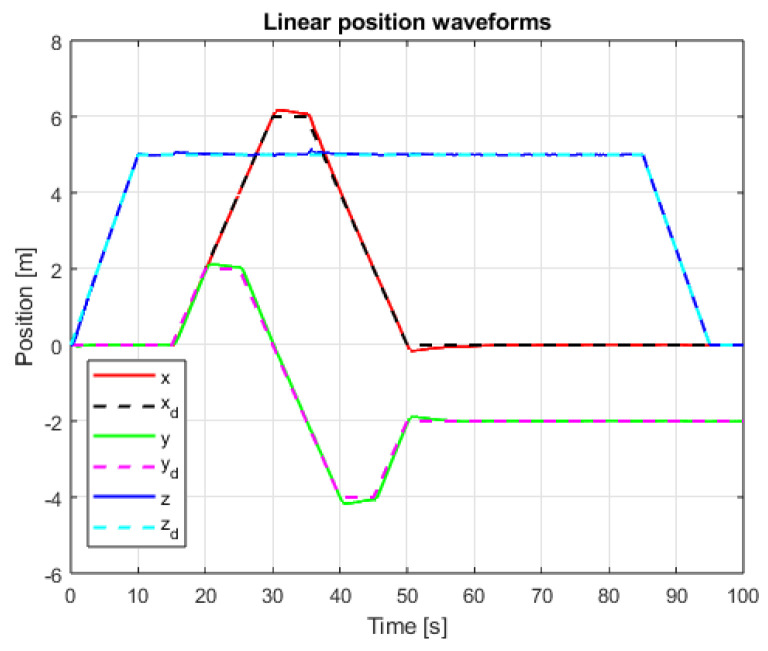
Waveforms of linear position signals and setpoint signals (PID+PID, interference gain: 1, delay: 0.001 s).

**Figure 12:**
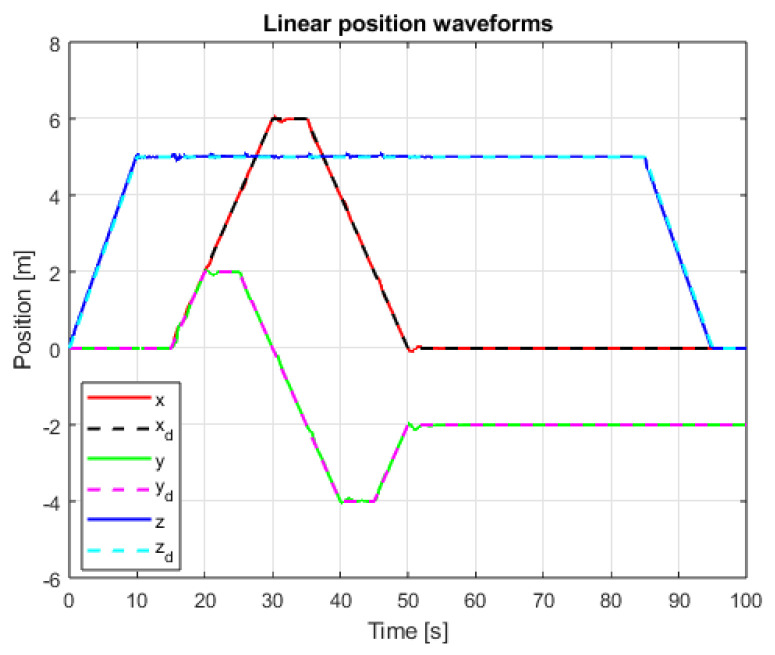
Waveforms of linear position signals and setpoint signals (SMC+SMC, interference gain: 1, delay: 0.001 s).

The results of linear positioning control system tests are visible in Figure 11 and Figure 12. The control task goals are accomplished for both used methods. In the case of PID cascade control system, noticeable overshoots are visible for X and Y axes’ positioning. Detailed observations and conclusions based on the measured values are presented in the next section.

**Figure 13:**
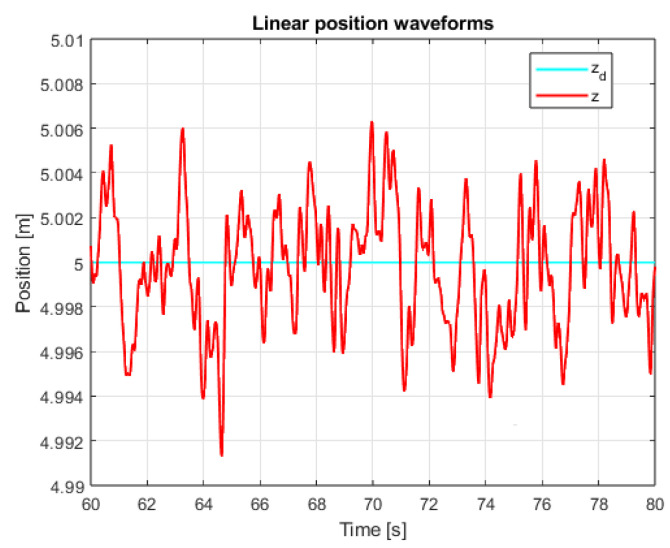
Waveforms of measured altitude and setpoint signal (PID+PID).

**Figure 14:**
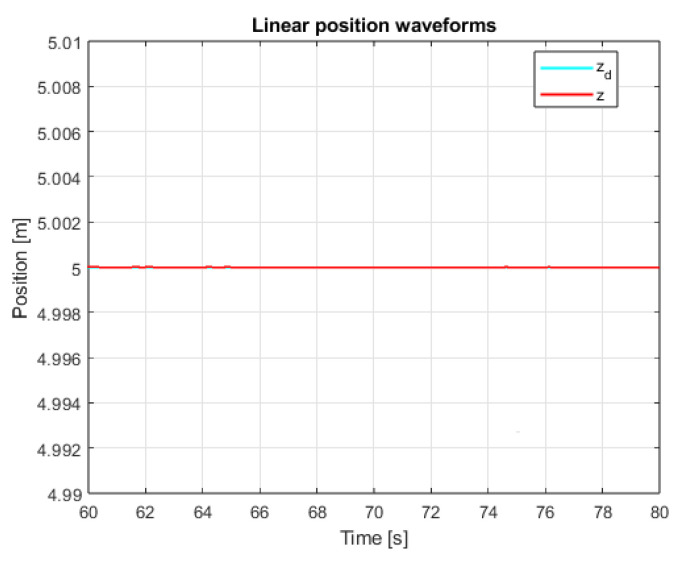
Waveforms of measured altitude and setpoint signal (SMC+SMC).

An additional, very important issue is the operation of the system in the steady state. This mainly refers to the resistance of the system to external disturbances, model inaccuracies and measurement noise. In order to verify the system in the steady state, additional control measures were measured. The linear position courses in the Z axis are presented in Figure 13 and Figure 14 for both controllers in the range of data allowing for noticing the disturbances and comparing them. The main observation based on these graphics leads to the conclusion that, according to the theory of Variable Structure Control systems, the sliding mode control algorithms provide system robustness to a wide range of disturbances.

## 9. Comparative Analysis of the Results of Simulation of Classic and Sliding Mode Control Systems

Selected control measures, which were used to examine the operation of individual controllers, are Integral of Squared Error (ISE) and Integral of Absolute Error (IAE).

Table 1 presents the values of control measures for both controllers in the case of angular positioning system tests. It is evident that the sliding mode controllers allow to improve the quality of the control process and provide greater robustness to applied disturbances. Increasing the gain of the interference signal has a significant impact on the operation of the PID controller. In the case of sliding mode controllers, the increase in this gain has a much smaller impact on the quality of the regulation.

**Figure 15:**
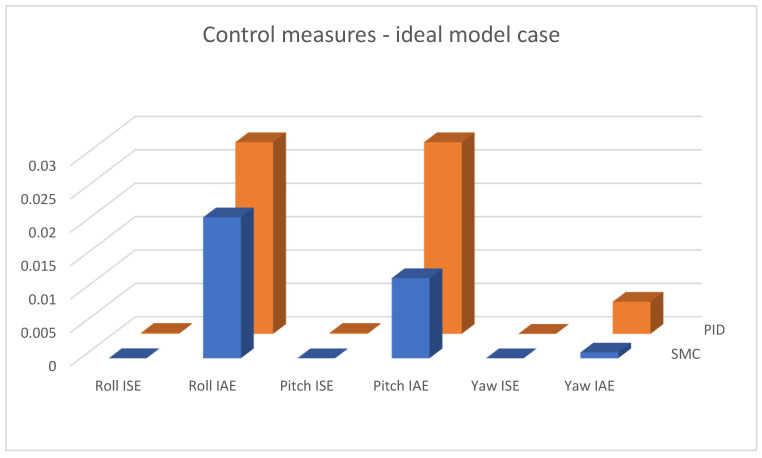
Bar charts presenting collected control measures—angular positioning case; ideal model used.

**Figure 16:**
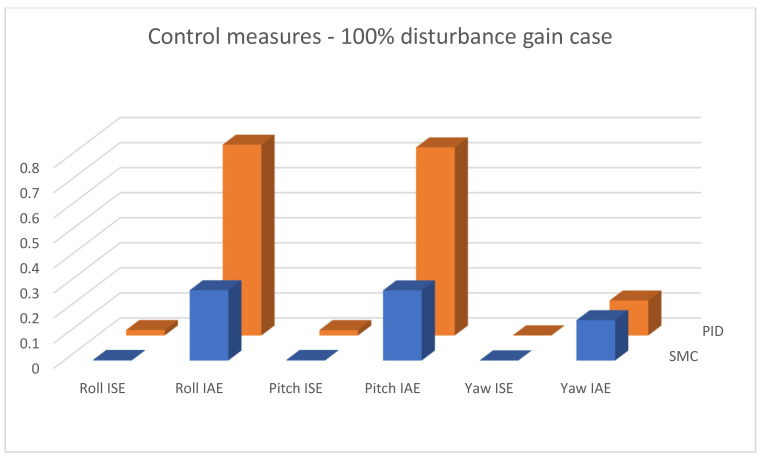
Bar charts presenting collected control measures—angular positioning case; disturbance applied (100% gain).

**Figure 17:**
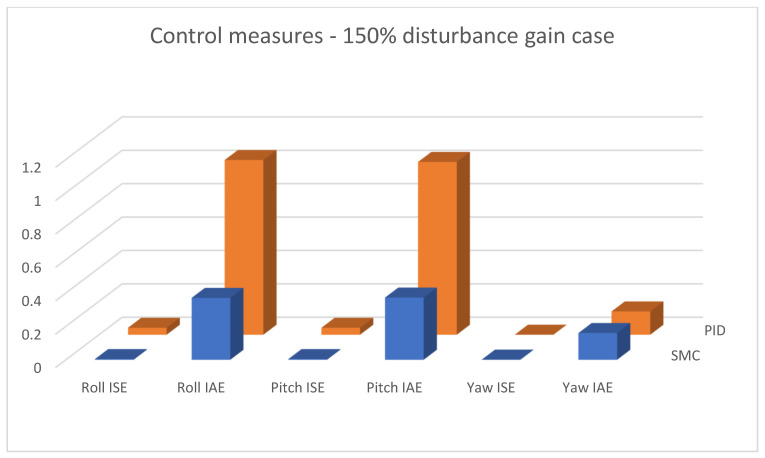
Bar charts presenting collected control measures—angular positioning case; disturbance applied (150% gain).

**Figure 18:**
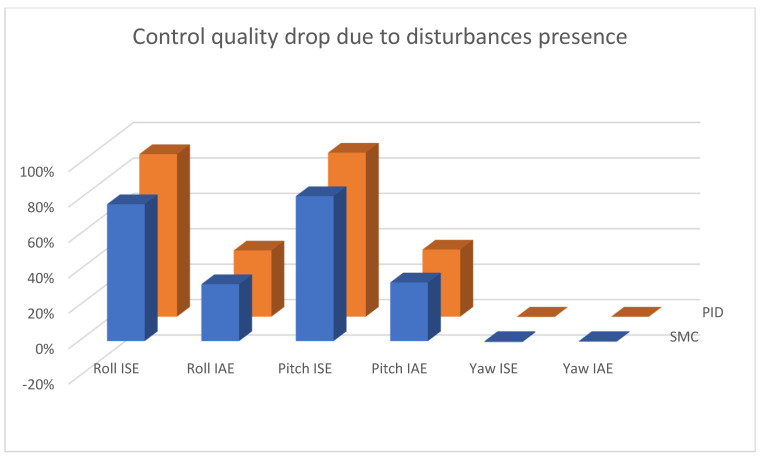
Bar charts presenting change in the control quality—angular positioning case; comparing two cases: 100% and 150% disturbance gains.

In Figure 15, Figure 16 and Figure 17, combined results of logging control measures is presented. One can see that sliding mode control algorithms ensure better control quality and, what is most important, provide the system with robustness to the applied disturbances, which becomes significantly more visible with the increase in the amplification of these disturbances. Figure 18 shows a percentage drop of control quality based on logged control measures. Sliding mode controllers, proposed in this paper, are burdened with a smaller negative impact of increased interference gain than classical methods based on PID controllers.

Table 2 presents logged control measures for the case of the linear positioning test. This table is analogous to Table 1 but contains an additional series of data which describe the system control quality for the steady state. The visualization of the collected data is presented below in the appropriate bar charts.

**Figure 19:**
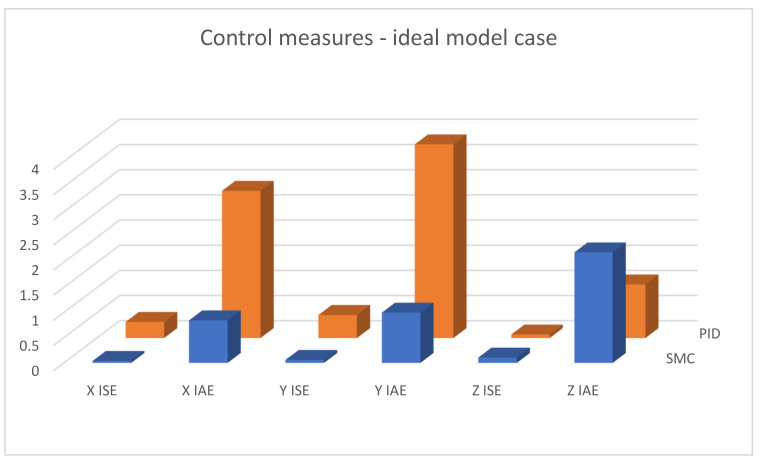
Bar charts presenting collected control measures—linear positioning case; ideal model used.

**Figure 20:**
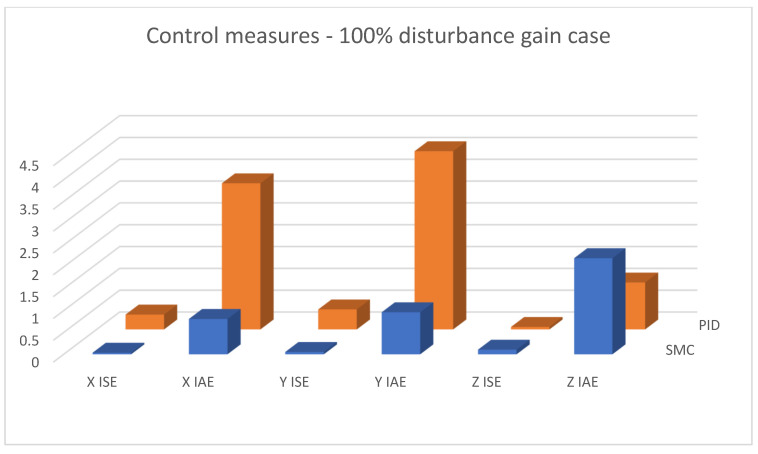
Bar charts presenting collected control measures—linear positioning case; disturbance applied (100% gain).

**Figure 21:**
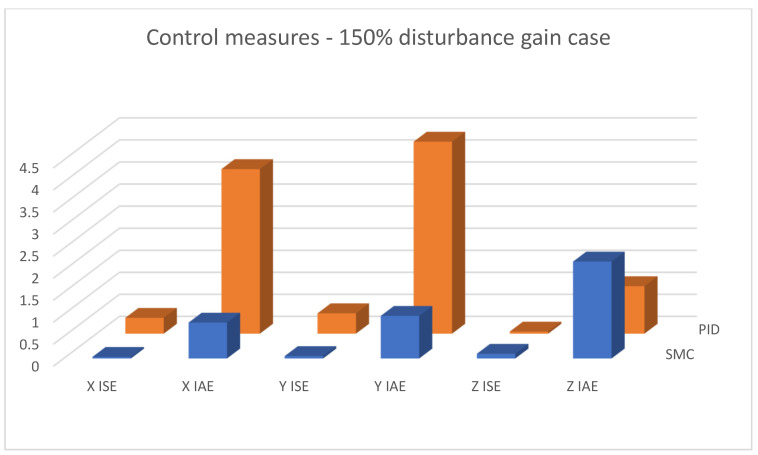
Bar charts presenting collected control measures—linear positioning case; disturbance applied (150% gain).

Figure 19, Figure 20 and Figure 21 shows linear positioning control system performance based on calculated control measures logged during simulation tests. It is clearly visible that the proposed sliding mode control algorithm allows for obtaining much better results in the case of X and Y linear position value control. However, the analysis of quality measures shows that the results for altitude control are visibly, although slightly, better for the case of classical PID controllers. This is due to the fact that the equivalent control of the altitude controller is also used as a component of the equivalent control of the X and Y linear position controllers; this causes the work of the algorithms to influence each other, which is visible in the form of small overshoots in dynamic states.

**Figure 22:**
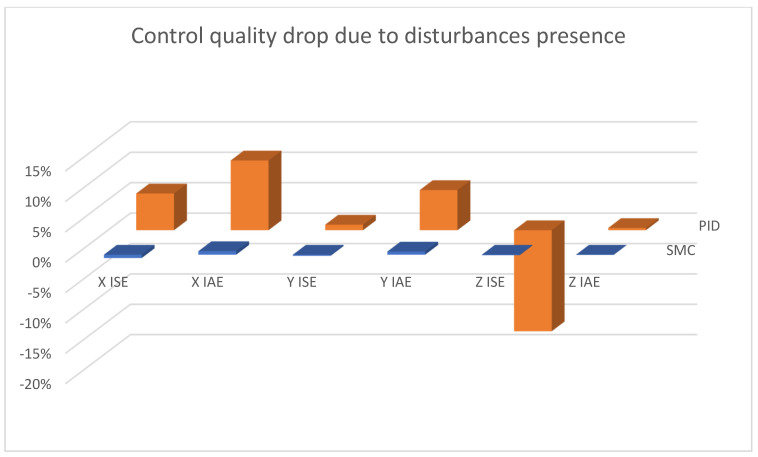
Bar charts presenting change in the control quality—linear positioning case; comparing two cases: 100% and 150% disturbance gains.

In Figure 22, one can see that the quality control does not deteriorate in any significant way in the case of sliding mode control, in contrast to PID control, for which the altitude control does not deteriorate either, but for X and Y linear position control, it becomes much less effective.

**Figure 23:**
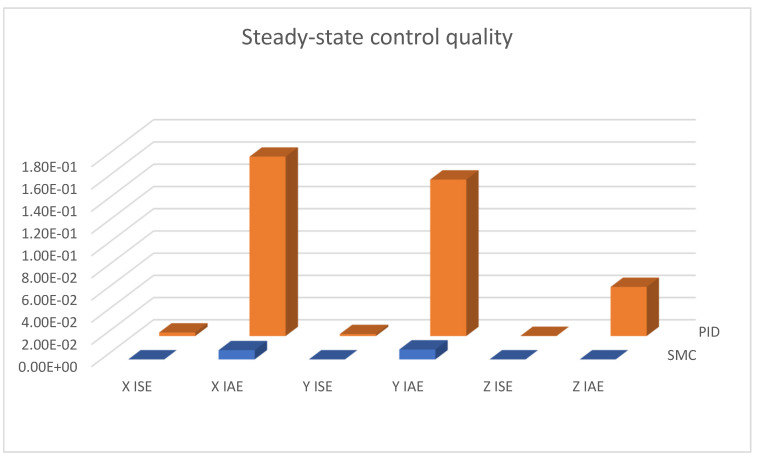
Bar charts presenting collected control measures—linear positioning steady-state case.

Due to the mentioned dependencies of equivalent control signals in the case of sliding mode control, the mentioned small amplitude overshoots in dynamic states appeared. Therefore, in order to fully test the quality of the regulation of the proposed controllers, quality measured for the steady-state test was collected. These data were measured after the quadcopter had previously risen to the set altitude. The measured data are presented in both Table 2 and Figure 23; it can be seen that, in accordance with the observations in Figure 13 and Figure 14, the sliding mode control algorithms provide significantly better results, especially by ensuring the system with robustness to external factors as well as model inaccuracies.

The remaining issue to be discussed is the influence of the introduced variable amplitude discontinuous control on the system, especially on the chattering phenomenon. Additional tests were performed to show the influence of the µ value on the presence of high-frequency oscillations caused by the use of the signum function in the control law. Control measures were collected for the steady-state case of the quadrotor linear position using different values of the µ parameter.

**Figure 24:**
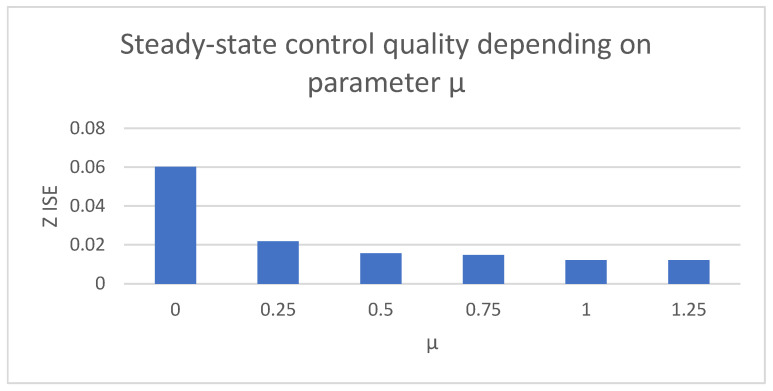
Bar charts presenting collected control measures—parameter µ influence.

Table 3 shows the control measures collected for the steady-state case of the quadcopter altitude signal. Depending on the used value of the µ parameter, it is possible to obtain significantly different results; for µ = 0, a case with classic discontinuous control with constant amplitude was obtained. Increasing the µ parameter leads to a decrease in the influence of chattering on the system. A comparison is shown in Figure 24. The higher the µ value, the better the quality of the control in the steady state. The waveforms presenting the chattering occurring on the quadcopter altitude signal are presented below.

**Figure 25:**
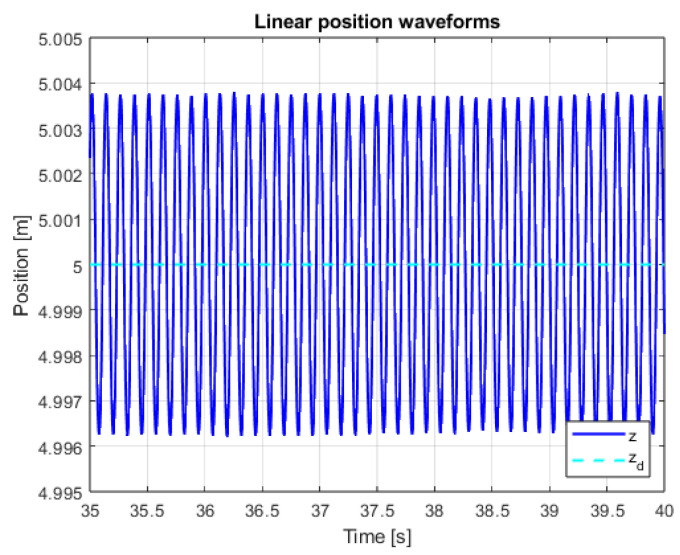
Waveforms of measured altitude and setpoint signal for µ = 0.

**Figure 26:**
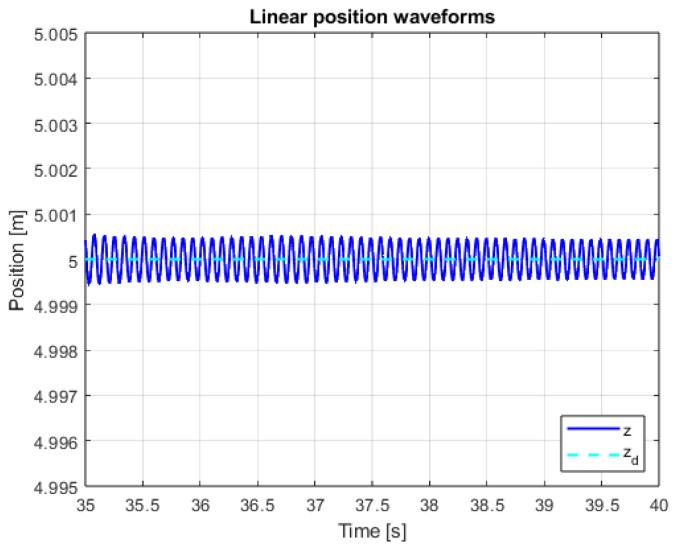
Waveforms of measured altitude and setpoint signal for µ = 0.25.

**Figure 27:**
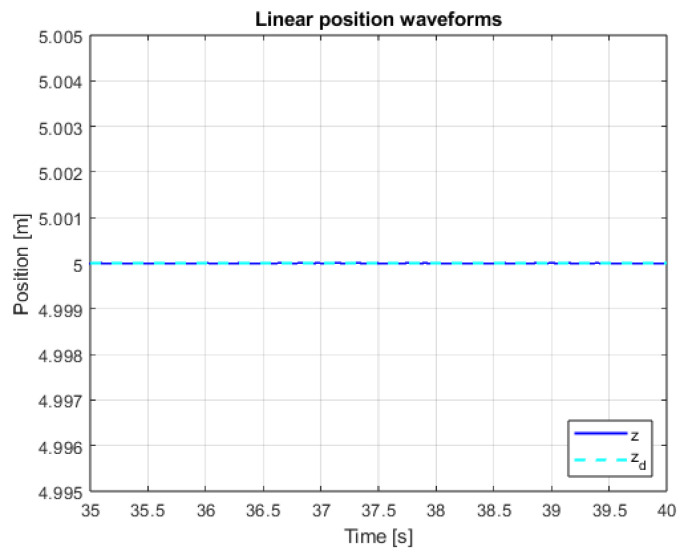
Waveforms of measured altitude and setpoint signal for µ = 1.

Figure 25, Figure 26 and Figure 27 presents quadcopter steady-state altitude and setpoint signal waveforms. Using classical approach of implementing discontinuous control with constant amplitude leads to visible chattering phenomenon occurrence. High-frequency oscillations related to the drone’s position are undesirable and can even be dangerous. Increasing the µ parameter leads to significant reduction in the impact of chattering, which is visible for cases when µ = 0.25 and µ = 1. The last mentioned value was used in the previous, main studies conducted for the purposes of this work (Figure 19, Figure 20, Figure 21, Figure 22 and Figure 23).

**Figure 28:**
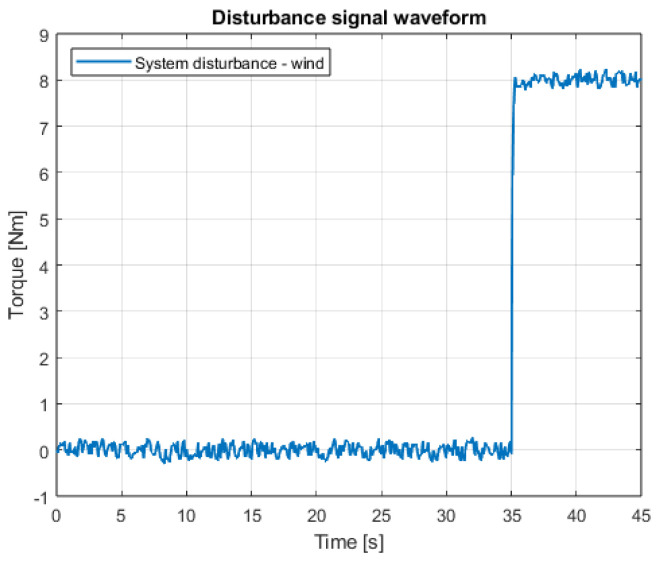
The waveform of the modeled external extreme interference signal.

**Figure 29:**
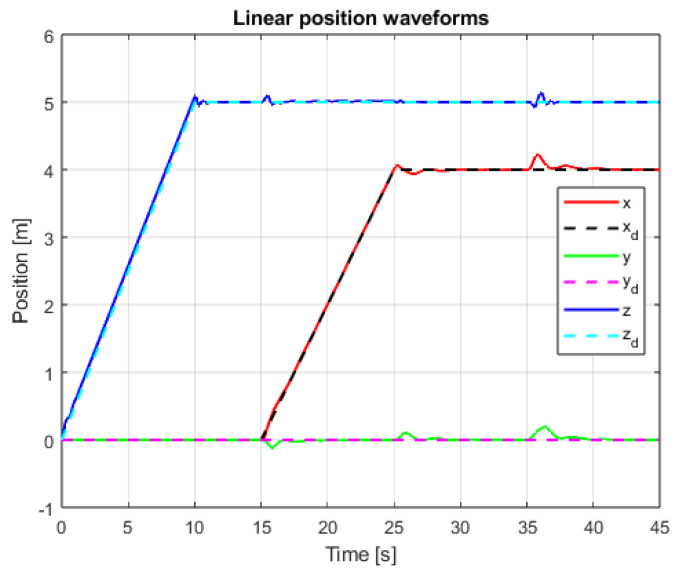
Waveforms of linear position signals and setpoint signals in case of extreme disturbance presence in the system.

Finally, to check whether the introduced changes do not deprive the system of its robustness related to the Variable Structure System property, an additional experiment was conducted, the aim of which was to introduce a strong, extreme disturbance. This disturbance (signal waveform presented in Figure 28) is a modeled strong wind, the value of which changes almost abruptly, which is to imitate the situation of a quadcopter leaving a wind shield (e.g., flying out from behind a building wall). In Figure 29, quadcopter linear positions are shown. It is very visible that the introduced extreme disturbance affects the system, but does not cause its stability to be lost. The control system quickly brings the position values back to the correct steady state. There were small and momentary overshoots, which are completely acceptable in the event of this type of disturbance. The proposed method is therefore highly robust to interference and, at the same time, ensures a significant reduction in the impact of the chattering phenomenon.

## 10. Conclusions

The presented method of synthesis of sliding mode controllers as a control system for a quadrotor achieves better results than classical controllers for both control structures: angular and linear positioning systems. It is largely based on the dynamics of the model; hence, it is necessary to estimate the parameters of the model with appropriate accuracy, which especially matters in the case of the outer loop control method, for which complex control laws are proposed. The presented results also confirm that SMC controllers better reduce the negative impact of external interference and delays in the measurement path, providing the control system with high level of robustness. The advantages of this method of control are more evident in the case of highly nonlinear objects, such as the discussed drones. Although classic control algorithms for quadrotor allow for achieving the expected results, it is worth developing new, progressive control methods, especially in the field of glide control.

## Figures and Tables

**Table 1:** Summary of the obtained values of control measures for each angular position control test case.

Types and Parameters of Controller		Ideal Model Case	Disturbance (100% Gain)	Disturbance (150% Gain)
PID	Roll ISE	0.000147570	0.021800000	0.041800000
	Roll IAE	0.028700000	0.761900000	1.046600000
	Pitch ISE	0.000147570	0.021600000	0.041600000
	Pitch IAE	0.028700000	0.750700000	1.035100000
	Yaw ISE	0.000003943	0.000479500	0.000478460
	Yaw IAE	0.004800000	0.139600000	0.139500000
SMC	Roll ISE	0.000012444	0.002200000	0.003900000
	Roll IAE	0.021100000	0.280500000	0.370600000
	Pitch ISE	0.000006852	0.002200000	0.004000000
	Pitch IAE	0.012000000	0.279900000	0.372500000
	Yaw ISE	0.000000089	0.000690170	0.000686050
	Yaw IAE	0.00086577	0.160800000	0.160200000

**Table 2:** Summary of the obtained values of control measures for each linear position control test case.

Types and Parameters of Controller		Ideal Model Case	Disturbance (100% Gain)	Disturbance (150% Gain)	Steady State
PID	X ISE	0.3206	0.3396	0.3601	0.0032
	X IAE	2.9261	3.3482	3.7319	0.1615
	Y ISE	0.4592	0.4585	0.4627	0.0018
	Y IAE	3.8488	4.0841	4.3535	0.1409
	Z ISE	0.0697	0.0571	0.0476	1.48 × 10^−4^
	Z IAE	1.0653	1.0744	1.0784	0.0443
SMC	X ISE	0.0394	0.0362	0.036	4.05 × 10^−6^
	X IAE	0.8465	0.8103	0.815	0.0083
	Y ISE	0.059	0.0547	0.0546	4.97 × 10^−6^
	Y IAE	1.0009	0.963	0.968	0.0089
	Z ISE	0.1061	0.1069	0.1068	4.16 × 10^−13^
	Z IAE	2.1999	2.202	2.2012	2.39 × 10^−6^

**Table 3:** Control measures of altitude control test case for different µ parameter values.

µ	Z ISE	Z IAE
0	0.000298533957762	0.060149331846856
0.25	0.000172976298346	0.021814947905653
0.5	0.000179834715996	0.015723003012009
0.75	0.000162966965424	0.014817696593609
1	0.000112808523373	0.012217696398422
1.25	0.000083358788050	0.012133886733481

## Data Availability

The data presented in this study are available on request from the corresponding author.
